# Supplementary material for: The role of reciprocity in human-robot social influence
Source: iScience. 2021 Nov 11;24(12):103424. doi: 10.1016/j.isci.2021.103424 (PMC8633024; doi:10.1016/j.isci.2021.103424)
Supplement: Document S1. Supplemental information models 1–6 and Tables S1–S6 [file mmc1.pdf]

iScience, Volume *24*

Supplemental information

The role of reciprocity

in human-robot social influence

Joshua Zonca, Anna Folsø, and Alessandra Sciutti

Supplemental information

We report the details of the models ran in the present work. For all models**,** the intercept was allowed to vary across participants including random effects at subject level.

In every model equation, β expresses coefficients of fixed effects, while u indicates random effects.

In the fixed-effect results, B express unstandardized regression coefficients. Independent categorical variables (condition and block, 2 levels) have been treated as dummy variables.

Social influence task

Model 1. Effect of response distance by group on participants' influence.

We tested the effect of the distance between the participant's estimate and the partner's one on trial-by-trial influence, depending on the experimental group (Computer or Robot). We used the following mixed-effects linear model:

$$I = \beta_0 + \beta_1 g + \beta_2 d + \beta_3 g * d + u_0 + \varepsilon$$

I is the participant's influence (i.e., susceptibility to the partner), g is the experimental group and d is the normalized distance between the two partner' responses (response distance (cm) / stimulus length (cm)). The Computer group is the reference category in the model output.

Reciprocal social influence task

Model 2: Effect of partner's influence level on performance ratings.

We tested the effect of the partner's mean influence (expressed in observation trials) on participants' performance ratings (self - other). We used the following mixed-effect linear model:

$$R = \beta_0 + \beta_1 i + u_0 + \varepsilon$$

R is the difference between participants' rating of own performance minus rating of partner's performance. These ratings are obtained across two different time points (at the end of the Susceptible block and the Unsusceptible block). i is the partner's influence, which refers to two specific time points (mean influence in the Susceptible block and in the Unsusceptible block). We ran the following mixed-effects linear model.

Model 3: Effect of condition by group on participants' estimation error.

We tested the effect of condition (Baseline, Susceptible or Unsusceptible) by experimental group (Computer or Robot) on participants' trial-by-trial estimation error. The Baseline condition corresponds to the Perceptual inference task, in which participants made perceptual estimates and final decisions, but did not have feedback about the partner's susceptibility. We focused on decision trials only, in order to guarantee the comparability with the Perceptual inference task (which is composed of decision trials) and with the subsequent analysis of participants' influence (which is expressed only in decision trials). We used the following mixed-effect linear model:

$$E = \beta_0 + \beta_1 g + \beta_2 c + \beta_3 g * c + u_0 + \varepsilon$$

E is the trial-by-trial estimation error (normalized by stimulus length), g is the experimental group (Computer or Robot), c is the experimental condition (Baseline, Susceptible or Unsusceptible). The Baseline condition and the Computer group are the reference categories in the model output.

Model 4: Effect of condition by group on response distance.

We tested the effect of condition (Baseline, Susceptible or Unsusceptible) by experimental group (Computer or Robot) on participants' trial-by-trial response distance (i.e., distance between the estimates of the two partners). The Baseline condition corresponds to the Perceptual inference task, in which participants made perceptual estimates and final decisions, but did not have feedback about the partner's susceptibility. We focused on decision trials only, in order to guarantee the comparability with the Perceptual inference task (which is composed of decision trials) and with the subsequent analysis of participants' influence (which is expressed only in decision trials). We used the following mixed-effect linear model:

$$D = \beta_0 + \beta_1 g + \beta_2 c + \beta_3 g * c + u_0 + \varepsilon$$

D is the trial-by-trial response distance (normalized by stimulus length), g is the experimental group (Computer or Robot), c is the experimental condition (Baseline, Susceptible or Unsusceptible). The Baseline condition and the Computer group are the reference categories in the model output.

<u>Model 5: Effect of condition by group on participants' influence.</u>

We tested the effect of condition (Baseline, Susceptible or Unsusceptible) by experimental group (Computer or Robot) on participants' trial-by-trial influence in decision trials. The Baseline condition corresponds to the Perceptual inference task, in which participants made perceptual estimates and final decisions, but did not have feedback about the partner's susceptibility to their judgments. We ran the following mixed-effect linear model:

$$I = \beta_0 + \beta_1 g + \beta_2 c + \beta_3 g * c + u_0 + \varepsilon$$

I is the trial-by-trial influence, g is the experimental group (Computer or Robot), c is the experimental condition (Baseline, Susceptible or Unsusceptible). The Baseline condition and the Computer group are the reference categories in the model output.

<u>Model 6: Effect of condition by group on participants' influence.</u>

We tested the effect of block (Final block, last block of the main task) by experimental group (Computer or Robot) on participants' trial-by-trial influence in decision trials. We ran the following mixed-effect linear model:

$$I = \beta_0 + \beta_1 g + \beta_2 c + \beta_3 g * c + u_0 + \varepsilon$$

I is the trial-by-trial influence, g is the experimental group (Computer or Robot), b is the experimental condition (Final block, last block of the main task). The block of the main task and the Robot group are the reference categories in the model output.

Supplemental Tables

Table S1. Results of Model 1. Related to Figure 3B.

Influence	B	Std. Err.	z	p	95% Conf. Interval	
Group (Robot – Computer)	0.047	0.041	1.16	0.246	- 0.032	0.127
Distance (Computer)	- 0.435	0.035	- 12.33	< 0.001	- 0.505	- 0.366
Group*Distance (Robot – Computer)	0.137	0.048	2.87	0.004	0.043	0.230
N. obs	3300					
N. groups	50					

Despite the significant interaction effect, revealing that the effect of distance was stronger in the Computer than in the Robot group, we report that also the Robot group shows a significant effect of distance (B = 0.299, SE = 0.032, z = - 9.36, p < 0.001, 95 % CI = [- 0.361, - 0.236]).

Table S2. Results of Model 2. Related to Results (Reciprocal social influence task).

Ratings (self – other)	B	Std. Err.	z	p	95% Conf. Interval	
Partner's influence	1.752	0.474	3.70	< 0.001	0.823	2.682
N. obs	100					
N. groups	50					

Table S3. Results of Model 3. Related to Figure 4.

Estimation error	B	Std. Err.	z	p	95% Conf. Inter.	
Group (Baseline condition)						
Robot - Computer	- 0.010	0.018	- 0.53	0.594	- 0.046	0.026
Condition (Computer group)						
Susceptible – Baseline	- 0.016	0.006	- 2.75	0.006	- 0.027	- 0.004
Unsusceptible – Baseline	- 0.015	0.006	- 2.67	0.008	- 0.026	- 0.004
Group*Condition (Robot – Computer)						
Susceptible – Baseline	0.032	0.008	4.00	< 0.001	0.016	0.048
Unsusceptible – Baseline	0.023	0.008	2.90	0.004	0.008	0.039
N. obs	6600					
N. groups	50					

We also report the effects of condition for the Robot group (Susc. – Baseline: B = 0.016, SE = 0.006, z = 2.91, p = 0.004, 95 % CI = [0.005, 0.028]; Unsusc. – Baseline: B = 0.008, SE = 0.006, z = 1.43, p = 0.154, 95 % CI = [- 0.003, 0.019].

Table S4. Results of Model 4. Related to Results (Reciprocal social influence task).

Response distance	B	Std. Err.	z	p	95% Conf. Inter.	
Group (Baseline condition)						
Robot - Computer	- 0.013	0.016	- 0.84	0.400	- 0.044	0.017
Condition (Computer group)						
Susceptible – Baseline	- 0.015	0.006	- 2.30	0.021	- 0.028	- 0.002
Unsusceptible – Baseline	- 0.022	0.006	- 3.42	0.001	- 0.035	- 0.009
Group*Condition (Robot – Computer)						
Susceptible – Baseline	0.033	0.009	3.61	< 0.001	0.015	0.051
Unsusceptible – Baseline	0.031	0.009	3.35	0.001	0.013	0.049
N. obs	6600					
N. groups	50					

We also report the effects of condition for the Robot group (Susc. – Baseline: B = 0.018, SE = 0.006, z = 2.80, p = 0.005, 95 % CI = [0.005, 0.031]; Unsusc. – Baseline: B = 0.009, SE = 0.006, z = 1.32, p = 0.188, 95 % CI = [- 0.004, 0.021].

Table S5. Results of Model 5. Related to Figure 4.

Influence	B	Std. Err.	z	p	95% Conf. Inter.	
Group (Baseline condition)						
Robot - Computer	0.084	0.040	2.12	0.034	0.006	0.162
Condition (Computer group)						
Susceptible – Baseline	0.026	0.009	2.95	0.003	0.009	0.043
Unsusceptible – Baseline	0.042	0.009	4.74	< 0.001	0.024	0.059
Group*Condition (Robot – Computer)						
Susceptible – Baseline	- 0.030	0.013	- 2.41	0.016	- 0.055	- 0.006
Unsusceptible – Baseline	- 0.029	0.013	- 2.31	0.021	- 0.053	- 0.004
N. obs	6600					
N. groups	50					

We also report the effects of condition for the Robot group (Susc. – Baseline: B = - 0.004, SE = 0.009, z = - 0.46, p = 0.644, 95 % CI = [- 0.021, 0.013]; Unsusc. – Baseline: B = 0.013, SE = 0.009, z = 1.48, p = 0.140, 95 % CI = [- 0.004, 0.030].

Table S6. Results of Model 6. Related to Results (Reciprocal social influence task).

Influence	B	Std. Err.	z	p	95% Conf. Inter.	
Group (Main task)						
Computer - Robot	- 0.052	0.044	- 1.19	0.232	- 0.139	0.034
Block (Robot group)						
Final - Main	- 0.034	0.014	- 2.45	0.014	- 0.061	- 0.007
Group*Block (Computer – Robot)						
Final - Main	0.018	0.020	0.93	0.354	- 0.020	0.056
N. obs	2200					
N. groups	50					

We also report the effects of block for the Computer group (Final - Main: B = - 0.016, SE = 0.014, z = - 1.14, p = 0.255, 95 % CI = [- 0.043, 0.011]).
